# It is all about perspectives—poor agreement between the patient, their parent and their physician in perception of spinal appearance in adolescent idiopathic scoliosis

**DOI:** 10.1007/s43390-026-01346-6

**Published:** 2026-04-04

**Authors:** Dineke G. van de Fliert, Tom P. C. Schlösser, Pepijn Bisseling, Marinus de Kleuver, Joost P. H. J. Rutges, Miranda L. van Hooff, Diederik H. R. Kempen

**Affiliations:** 1 Department of Orthopedic Surgery, Radboud University Medical Center, Nijmegen, The Netherlands; 2 Department of Orthopedic Surgery, University Medical Center Utrecht, Utrecht, The Netherlands; 3 Department of Orthopedic Surgery, Sint Maartenskliniek, Nijmegen, The Netherlands; 4 Department of Orthopaedics & Sports Medicine, Erasmus MC, Rotterdam, The Netherlands; 5 Department of Research, Sint Maartenskliniek, Nijmegen, The Netherlands; 6 Department of Orthopedic Surgery, OLVG Amsterdam, Amsterdam, The Netherlands

**Keywords:** Scoliosis, Spinal appearance questionnaire (SAQ), Physical appearance, Proxy

## Abstract

**Purpose:**

Adolescent idiopathic scoliosis (AIS) affects the appearance of the trunk and spine. The Spinal Appearance Questionnaire (SAQ) measures the AIS patients’ perception of appearance, but may be challenging for patients, because parts of the trunk changes are not visible to themselves (e.g. posterior rib hump). Perceptions may therefore differ between patients, parents and physicians. The study aim is to investigate whether perceptions of physicians and parents align with the patient’s perception of their appearance, as measured with the SAQ.

**Methods:**

The SAQ (Dutch) was administered to 108 AIS patients (15.3 years [SD 2.2], 77.8% girls), their parent and physician (*n* = 15). Parents completed it twice, based on their own perception (parent’s perspective) and their conceived child’s perception (parent-patient’s perspective). For all four perspectives, the 10 individual items and total appearance domain scores were compared.

**Results:**

The appearance domain scores differed not between the four perspectives (*p* = 0.248) and the ICCs compared with the patient’s own scores were 0.63 (95%CI 0.47–0.75) for the parent’s perspective, 0.60 (95%CI 0.45–0.71) for the parent-patient’s perspective and 0.44 (95%CI 0.27–0.58) for the physician’s perspective. The ICC per item varied from − 0.01 to 0.64.

**Conclusion:**

For the appearance domain score, moderate agreement was found between the patient’s, and their parent’s and parent-patient’s perspectives. Poor agreement was found between the patient’s and physician’s perspectives. For the 10 items, poor to moderate agreement was found between the patient and their parents or physicians. During scoliosis treatment, physicians should be aware of differences in perceptions of appearance of patients, parents and themselves.

## Introduction

Adolescent idiopathic scoliosis (AIS) is a three-dimensional deformity of the spine and trunk. It develops during their adolescence, a sensitive period of life when adolescence may experience social, emotional, cognitive and physical changes and developments [1, 2]. By progression of the deformation of the trunk, AIS patients can suffer from changes in their cosmetic appearance. These physical changes can have a significant emotional and psychological impact, potentially leading to a decreased self-image, particularly in adolescent patients. Both parents and physicians may find it challenging to discuss on the impact of physical appearance with the adolescents due to its sensitive nature.

The Spinal Appearance Questionnaire (SAQ) questionnaire has been developed to assess the perception of AIS patients of their physical appearance [3, 4]. It has been translated into more than 10 languages [5–16], and its measurement properties have been evaluated. The appearance domain consists of 10 items in which the patient can score the severity of their trunk and spinal deformities. The scoring is based on a Walter Read Visual Assessment Scale, using five illustrations with varying severities of the spine and trunk deformity [3]. Because no references are provided, patients have no reference values or illustrations. Subsequently, the SAQ is a subjective questionnaire focusing on the patients’ own perspective.

Conversations about the patients’ perspective of their physical appearance can be a delicate topic during adolescence for parents and physicians. Since not all scoliosis characteristics are always visible for patients (e.g. rib hump on the back), the perception of these scoliosis related trunk changes might vary between patients, parents and physicians. Therefore, the aim of this study is to investigate whether perceptions of physicians and parents align or differ with the patient’s perception of their appearance, as measured with the SAQ.

## Methods

### Design and study population

For this cross-sectional multicenter study, consecutive patients in two academic and two non-academic centers caring for AIS patients were included. To minimize patient burden, we reached out to parents and the patients’ physician of 113 included AIS patients from a previous Dutch Spinal Appearance Questionnaire (SAQ) validation study [7]. AIS patients between 10 and 21 years old, that could read the Dutch language, with all types of scoliosis treatment (including observation, brace treatment and surgery) were included. On the same day at the outpatient clinic, the parents and the patients’ physician were also asked to participate in the study. All participants provided informed consent. This study was approved by the local Medical Ethical Committee (file number: 2021–13280) and local hospital review boards.

### Data collection

The study data was collected with Castor EDC (Electronic Data Capture) [17]. The patients, parents and physicians were asked to complete the appearance domain of the SAQ and questions concerning baseline characteristics. For the patients, the baseline characteristics included gender, age, maximum Cobb angle and maximum angle of trunk rotation (measured with a Bunnell Scoliometer). Parents were asked about their gender and family history on scoliosis. Gender was recorded for the treating physicians. No proxy versions of the SAQ were developed for the study.

The Dutch SAQ appearance domain consists of ten items, scored from 1 (best) to 5 (worst), whereby the final total score ranges from 10 to 50 [7]. The Dutch SAQ, including the appearance domain and the expectations domain, is shown in Supplementary file 1. It is an adequate, reliable and valid questionnaire to evaluate the AIS patients’ perception of appearance [7]. The AIS patients completed the questionnaire by themselves (*patient’s perspective*) for the validation study of the Dutch SAQ [7]. One of the parents that accompanied the patient was asked to participate in the study and to complete the questionnaire twice, independently and separately from their child. First, they were instructed to complete the SAQ from their own perspective (*parent’s perspective*). Second, they were instructed to complete the SAQ as they thought that their child perceives their appearance (*parent-patient’s perspective*). The treating physician (*physician’s perspective*) also completed the questionnaire regarding that patient immediately after the outpatient clinic appointment, ensuring that the appointment did not take any longer.

### Statistical analysis

Descriptive statistics were used to describe the study data. For categorical variables, the numbers (n) and percentages (%) were calculated. The Kolmogorov–Smirnov test was used to assess the distribution of the study variables. Normally distributed continuous data were described in means and standard deviations. In case of non-normality, median and ranges will be used. Missing data of baseline characteristics were, if present, clearly described.

For all four perspectives (patient, parent, parent-patient and physician), the total appearance domain scores of the SAQ were calculated. The level of agreement between the patient’s perspective and the three other perspectives (parent, parent-patient and physician) was evaluated with intraclass correlation coefficients (ICC: two-way mixed effects, single measurement, absolute agreement) per item and for the appearance domain score. The ICCs between the parent's and parent-patient's perspectives were also evaluated per item and for the appearance domain score. An ICC of less than 0.50 indicates poor reliability, an ICC between 0.50 and 0.75 is indicative of moderate reliability, an ICC between 0.75 and 0.90 is indicative of good reliability and an ICC of more than 0.90 indicates excellent reliability [18]. The Kruskal–Wallis test was used to compare the appearance domain scores between the four perspectives. To investigate the correlation between the angle of trunk rotation and the SAQ, the Spearman’s rank correlation analysis was used. The strength of this correlation is interpreted as “weak” (r = 0.10–0.30), “moderate” (r = 0.31–0.50), or “strong” (r = 0.51–1.00) [19]. All statistical analyses were performed in SPSS (IBM® SPSS® Statistics version 29). A *p*-value < 0.05 was considered statistically significant.

## Results

**Fig. 1:**
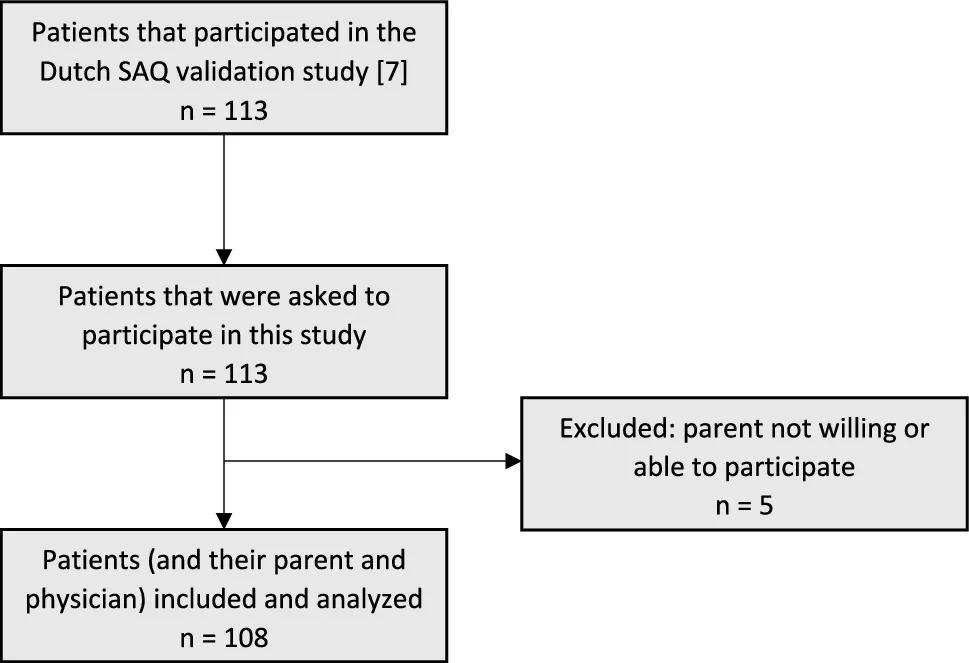
STROBE flow chart of the study participants. (SAQ Spinal Appearance Questionnaire, n number)

Of the 113 AIS patients that participated in the Dutch SAQ validation study, 5 parents were not willing (time shortage) or not able to (language barrier) complete the questionnaire. A total of 108 AIS patients, their parent and physician were included in this study (Fig. 1). All participants completed the questionnaires without missing items. The baseline characteristics are shown in Table 1. In total, 84 (77.8%) of the included AIS patients were female. The mean age of the patients was 15.3 years (SD 2.2) and the median maximum Cobb angle was 25 degrees (range 10°–68°). The majority of the parents that participated in the study were female (68.5%). Thirteen parents were also diagnosed with scoliosis themselves. In total 15 different physicians, of whom four women (26.7%), completed the questionnaires about their patient.

**Table 1:** Baseline characteristics of the study participants

	Variables	Values
Patient (*n* = 108)	Female gender	77.8% (*n* = 84)^a^
	Mean age	15.3 (± 2.2)^b^
	Maximum Cobb angle	25.0 (10.0–68.0)^c^
	Maximum Angle of Trunk Rotation	9.0 (1.0–23.0)^c^
Parent (*n* = 108)	Female gender	68.5% (*n* = 74)^a^
	Diagnosed with scoliosis	12.0% (*n* = 13)^a^
Physician (*n* = 15)	Female gender	26.7% (*n* = 4)^a^

^a^Percentage (number)

^b^Mean (SD)

^c^Median (range)

**Fig. 2:**
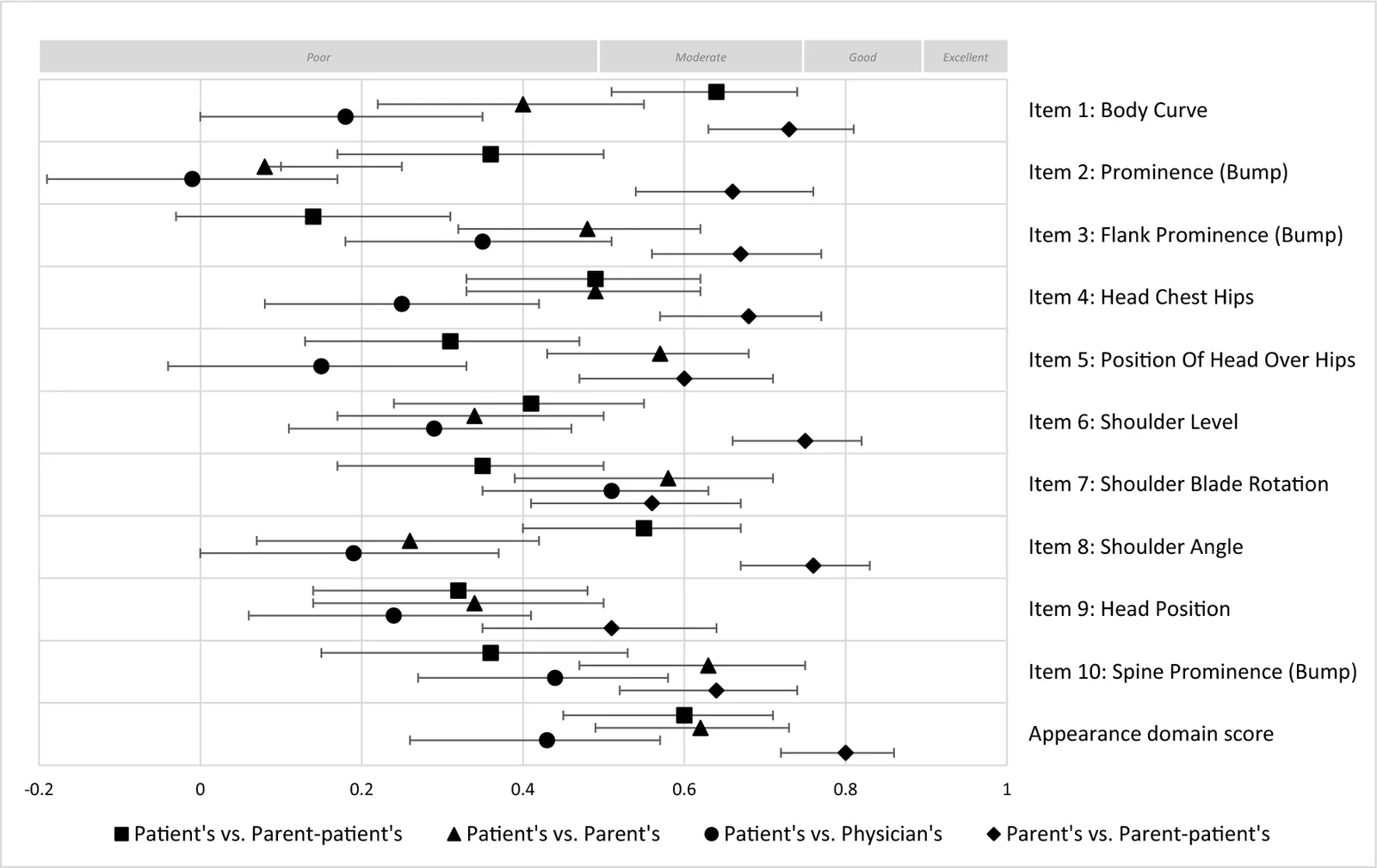
Overview of the Intraclass Correlation Coefficient (ICC) between the parents’ or physician’s perspectives and the patient’s perspective, and between the parent’s and parent-patient’s perspective per item and for the appearance domain in total. (The markers (square, triangle, circle and diamond) shows the ICC and the error bars show the 95% confidence interval)

**Fig. 3:**
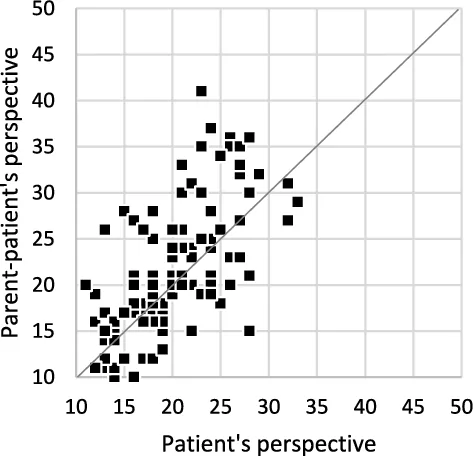
The scatter plot shows the appearance domain scores for the parent-patient’s perspective and the patient’s perspective for each patient (square). (The minimal score of the Appearance domain is 10 and the maximum score is 50. ICC = 0.60 (0.45–0.71))

**Fig. 4:**
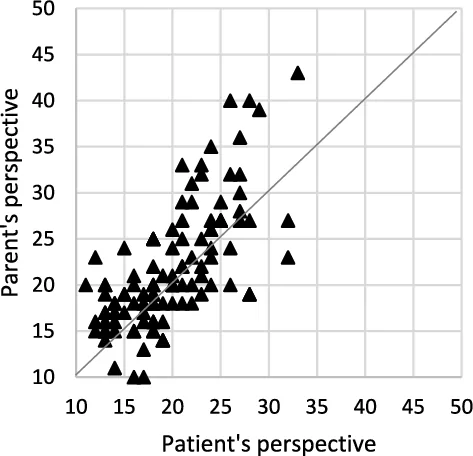
The scatter plot shows the appearance domain scores for the parent’s perspective and the patient’s perspective for each patient (triangle). (The minimal score of the Appearance domain is 10 and the maximum score is 50. ICC = 0.63 (0.47–0.75))

**Fig. 5:**
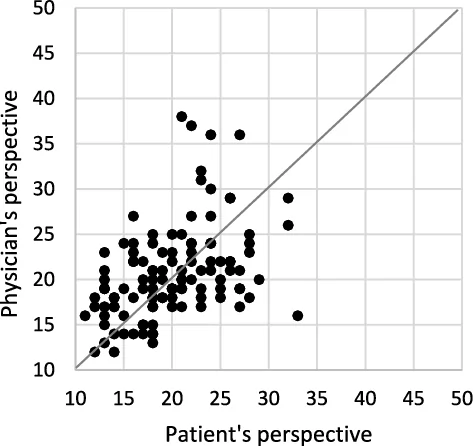
The scatter plot shows the Appearance domain scores for the physician’s perspective and the patient’s perspective for each patient (circle). (The minimal score of the Appearance domain is 10 and the maximum score is 50. ICC = 0.44 (0.27–0.58))

**Fig. 6:**
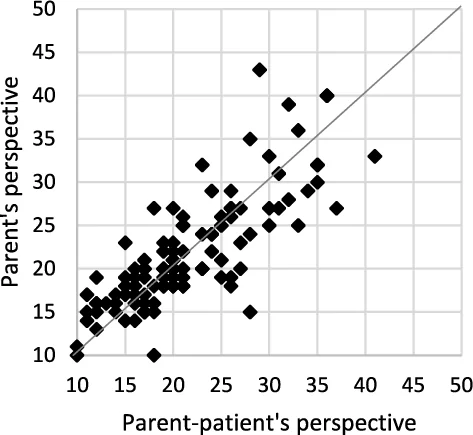
The scatter plot shows the appearance domain scores for the parent’s and parent-patient's perspective for each patient (diamond). (The minimal score of the Appearance domain is 10 and the maximum score is 50. ICC = 0.80 (0.72–0.86))

Table 2 shows the median scores per item of the SAQ appearance domain and for the appearance domain. The median appearance domain scores were all approximately 20, ranging from 11 to 33 for the patient’s, from 10 to 41 for the parent-patient’s, from 10 to 43 for the parent’s, and from 12 to 38 for the physician’s perspective. These median appearance domain scores were not significantly different between the four perspectives (χ2(3) = 4.133, *p* = 0.248). The correlation between the parent’s, parent-patient’s and physician’s perspectives and the patient’s perspective per item are shown in Table 3 and Fig. 2 and vary between an ICC of − 0.01 and an ICC of 0.64. Lowest correlations were found for item 3 about flank prominence (bump). For the total appearance domain, the ICC (compared with the patient’s perspective) was 0.60 (95%CI 0.45–0.71) for the parent-patient’s perspective, 0.63 (95%CI 0.47–0.75) for the parent’s perspective and 0.44 (95%CI 0.27–0.58) for the physician’s perspective (as shown in Table 3 and Fig. 2). The agreement of the appearance domain scores are visualized in scatter plots, between the patient’s perspective compared to the parent-patient’s (Fig. 3), parent’s (Fig. 4) and physician’s perspective (Fig. 5) for each patient. The ICC between parent’s and parent-patient’s perspective was 0.80 (95%CI 0.72–0.86), as visually represented in Fig. 6. Although the linear association between the perspectives is highlighted, the individual data points are scattered both above and below the ideal correlation line.

**Table 2:** Scores per perspective for each item and the appearance domain score of the SAQ

	Patient’s perspective	Parent-patient’s perspective	Parent’s perspective	Physician’s perspective
Item 1: Body curve	2 (1 – 5)^a^	2 (1 – 5)^a^	2 (1 – 5)^a^	2 (1 – 5)^a^
Item 2: Prominence (Bump)	2 (1 – 4)^a^	2 (1 – 4)^a^	2 (1 – 4)^a^	2 (1 – 5)^a^
Item 3: Flank prominence (Bump)	2 (1 – 4)^a^	2 (1 – 4)^a^	2 (1 – 4)^a^	2 (1 – 5)^a^
Item 4: Head chest hips	2 (1 – 5)^a^	2 (1 – 5)^a^	2 (1 – 5)^a^	2 (1 – 4)^a^
Item 5: Position of head over hips	2 (1 – 5)^a^	2 (1 – 4)^a^	2 (1 – 5)^a^	1 (1 – 4)^a^
Item 6: Shoulder level	2 (1 – 4)^a^	2 (1 – 4)^a^	2 (1 – 4)^a^	2 (1 – 4)^a^
Item 7: Shoulder blade rotation	2 (1 – 5)^a^	2 (1 – 5)^a^	2 (1 – 5)^a^	2 (1 – 4)^a^
Item 8: Shoulder angle	2 (1 – 4)^a^	2 (1 – 5)^a^	2 (1 – 5)^a^	2 (1 – 5)^a^
Item 9: Head position	2 (1 – 5)^a^	2 (1 – 5)^a^	2 (1 – 5)^a^	2 (1 – 5)^a^
Item 10: Spine prominence (Bump)	2 (1 – 4)^a^	2 (1 – 5)^a^	2 (1 – 5)^a^	2 (1 – 4)^a^
Appearance domain score^b^	19.5 (11.0 – 33.0)^a^	20.0 (10.0 – 41.0)^a^	20.0 (10.0 – 43.0)^a^	19.5 (12.0 – 38.0)^a^

Range of the scores per item: 1 – 5; Range of the scores for the appearance domain (10 – 50)

^a^Median (range)

^b^Kruskall–Wallis test (χ2(3) = 4.133, *p* = 0.248)

**Table 3:** Intraclass correlation coefficient between the parents’ or physician’s perspectives and the patient’s perspective, and between the parent’s and parent-patient’s perspective per item and for the appearance domain in total.

	Patient’s vs. parent-patient’s perspective	Patient’s vs. parent’s perspective	Patient’s vs. physician’s perspective	Parent’s vs. parent-patient’s perspective
Item 1: Body Curve	0.64 (0.51 – 0.74)^a^	0.62 (0.49 – 0.73)^a^	0.43 (0.26 – 0.57)^a^	0.73 (0.63 – 0.81)^a^
Item 2: Prominence (Bump)	0.36 (0.17 – 0.50)^a^	0.40 (0.22 – 0.55)^a^	0.18 (0.00 – 0.35)^a^	0.66 (0.54 – 0.76)^a^
Item 3: Flank prominence (Bump)	0.14 (− 0.03 – 0.31)^a^	0.08 (− 0.10 – 0.25)^a^	− 0.01 (− 0.19 – 0.17)^a^	0.67 (0.56 – 0.77)^a^
Item 4: Head chest hips	0.49 (0.33 – 0.62)^a^	0.48 (0.32 – 0.62)^a^	0.35 (0.18 – 0.51)^a^	0.68 (0.57 – 0.77)^a^
Item 5: Position of head over hips	0.31 (0.13 – 0.47)^a^	0.49 (0.33 – 0.62)^a^	0.25 (0.08 – 0.42)^a^	0.60 (0.47 – 0.71)^a^
Item 6: Shoulder level	0.41 (0.24 – 0.55)^a^	0.57 (0.43 – 0.68)^a^	0.15 (-0.04 – 0.33)^a^	0.75 (0.66 – 0.82)^a^
Item 7: Shoulder blade rotation	0.35 (0.17 – 0.50)^a^	0.34 (0.17 – 0.50)^a^	0.29 (0.11 – 0.46)^a^	0.56 (0.41 – 0.67)^a^
Item 8: Shoulder angle	0.55 (0.40 – 0.67)^a^	0.58 (0.39 – 0.71)^a^	0.51 (0.35 – 0.63)^a^	0.76 (0.67 – 0.83)^a^
Item 9: Head position	0.32 (0.14 – 0.48)^a^	0.26 (0.07 – 0.42)^a^	0.19 (0.00 – 0.37)^a^	0.51 (0.35 – 0.64)^a^
Item 10: Spine prominence (Bump)	0.36 (0.15 – 0.53)^a^	0.34 (0.14 – 0.50)^a^	0.24 (0.06 – 0.41)^a^	0.64 (0.52 – 0.74)^a^
Appearance domain score	0.60 (0.45 – 0.71)^a^	0.63 (0.47 – 0.75)^a^	0.44 (0.27 – 0.58)^a^	0.80 (0.72 – 0.86)^a^

^a^ Intraclass correlation coefficient (95% confidence interval), Two-way random effects, single measurement, for absolute agreement

Table 4 shows the correlations between the angle of trunk rotation and the scores for item 2 about prominence (bump) and item 3 about flank prominence (bump) and the appearance domain score. A strong correlation was found between the angle of trunk rotation and the physician’s score of item 2 and between the angle of trunk rotation and the physician’s appearance domain score.

**Table 4:** Correlation per perspective between the Angle of Trunk Rotation and scores for item 2 and 3 and for the appearance domain of the SAQ

	Patient’s perspective	Parent-patient’s perspective	Parent’s perspective	Physician’s perspective
Item 2: Prominence (Bump)	0.33 (*p* < 0.001)^a^	0.37 (*p* < 0.001)^a^	0.49 (*p* < 0.001)^a^	0.55 (*p* < 0.001)^a^
Item 3: Flank prominence (Bump)	0.27 (*p* < 0.05)^a^	0.20 (*p* < 0.05)^a^	0.08 (*p* = 0.395)^a^	0.22 (*p* < 0.05)^a^
Appearance domain score	0.49 (*p* < 0.001)^a^	0.43 (*p* < 0.001)^a^	0.47 (*p* < 0.001)^a^	0.60 (*p* < 0.001)^a^

^a^ Correlation coefficient (p-value) of the Spearman’s rank test

## Discussion

This study has shown that in this cross-sectional cohort of adolescents with an idiopathic scoliosis of varying magnitude, despite reasonable overall agreement between patients’, parents’ and physicians’ perspectives of appearance at a group level, there are very large individual differences in perception of appearance between patients, parents and physicians. Parents and especially physicians cannot serve as a proxy for the patient’s own perspective. Although this may not seem surprising, perception of appearance is a significant driver of patients’ expectations, compliance with (brace) treatment, and or choice of therapy, especially surgery. The moderate and poor agreement between patient’s and parent’s perspectives, and patient’s and physician’s perspectives highlight that no assumptions should be made in what the child thinks or feels and appearance should be a topic of discussion during the treatment.

Previous studies have compared the differences in perception of appearance using different questionnaires. So far, most studies have investigated how appearance is perceived before and after surgical correction of the scoliosis. Using the Quality of Life Profile for Spinal Disorders questionnaire, Smith et al. [20] found that radiographic and physical measures of the cosmetic deformity (such as rib prominence measured with a scoliometer) did not correlate well with the patients’ and parents’ perceptions of appearance. Furthermore, patients had an overall worse perception of their appearance compared to their parents [20]. After AIS surgery, patients and parents did not strongly agree on the cosmetic outcome of the procedure [20]. In a study by Sanders et al.[3], parents’ scores were significantly worse than those of patients for the trunk shift, kyphosis, prominence, shoulders and curve domains of the SAQ before surgery. Furthermore, orthopedists’ ratings of scar appearance, cosmetic deformity, and preoperative to postoperative cosmetic change did not significantly correlate with patient satisfaction [21]. To our knowledge, this is the first study to compare the appearance domain score and items scores of the SAQ between patients, their parents and their physicians across a broad range of curves. As with previous studies, the moderate and poor correlations between parents’ and patients’ ratings and between physicians’ and patients’ ratings in our study highlights the subjective nature of appearance perception, which depends on each patient’s individual opinion of aesthetics.

Parents may perceive the impact of their child’s disease differently from their child, and they may have a different view of its overall impact on their child’s life. By asking the parents to complete the questionnaire twice (parent-patient’s and parent’s perspective), their own perception was investigated as well as their understanding of how their child sees themselves. By comparing the scores of the patient’s perspective with the parent-patient’s perspective, poor to moderate agreement was found for the ten items (ICC varied from 0.14 to 0.64), implicating that parents do not always know how their child perceives their physical appearance. Poor to moderate agreement was also found between the patient’s and the parent’s perspective (ICC varied from 0.08 to 0.62) and patient’s and physician’s perspective (ICC varied from − 0.01 to 0.51). To rule out the possibility that parents simply filled out the SAQ twice the same, the ICCs between the two parent versions were examined. The ICC of 0.80 (and not 1.0) between the parent’s perspective and the parent-patient’s perspective, implies that the parent completed the two questionnaires differently and that they may have tried to put themselves in their child’s shoes. It might also imply that the parents themselves also think that they perceive the physical appearance different from their child.

The impact of AIS on appearance and thereby for example the extent of protruding ribs or loins varies per patient. The Bunnell Scoliometer is used to measure the ATR. For item 2 and item 3 of the SAQ, the patient scores the amount of their (rib) prominence and flank prominence, respectively. The correlation between the angle of trunk rotation and the score item 2 and 3 of the patients’, parents’ and parent-patients’ perspectives, varies from weak to moderate. A strong correlation was found between the physicians’ score on item 2 of the SAQ and the ATR, and a weak correlation was found between item 3 and the ATR. The physicians measured the ATR themselves at the appointment, but the answer options only show different severities of the possible prominences (without e.g., reference values in degrees) to the person that completes the questionnaire. Although the physician may have developed a frame of reference by seeing many AIS patients at the (outpatient) clinic, they do not score the appearance domain structurally lower (or minimize the appearance) and also score sometimes higher than the patients themselves (Fig. 5).

The SAQ is originally developed to evaluate the patients’ perception of appearance. The questionnaire, however, also contains items about the dorsal side of their trunk or in forward bending position. During testing the prefinal version of the Dutch SAQ, more than half of the patients mentioned that they weren’t aware of their trunk and spine from all angles, indicating limitation in their self-perception of appearance. The lowest correlations between patient’s perspective and the perspectives of the parents and physicians were found for item 3, which includes illustrations about flank prominence (bump) in forward bending position, an example of a deformity that is not readily visible to the patient. For this item, poor correlations were found between the patient’s perspective and the perspectives of the parents and physician (ICC ranging from − 0.01 to 0.14). Nonetheless, poor to moderate agreement was also found between the patient’s perspective and the parent’s and physician’s perspectives for other items of the SAQ, emphasizing that they all perceive the appearance of the patient differently. It has to be noted that a disadvantage of using the appearance domain score is that the total score for a patient and physician may be the same, even though the scores for each item may differ completely.

## Conclusion

Parents and physicians cannot serve as a proxy for patients with AIS when completing the SAQ, because their perceptions of physical appearance differ from those of the patients themselves, even when the parents were asked to fill in the questionnaire as they believe the patients see themselves. For the total appearance domain score of the SAQ, moderate agreement was found between the patient’s, and their parent’s and parent-patient’s perspectives. Poor agreement was found between patient’s and the physician’s perspectives. When comparing the ten individual items scores between the patient and their parents or physicians, poor to moderate agreement was found. During treatment of scoliosis, physicians should be aware of the differences in theirs and the patient’s perception of appearance and the differences in the patient’s and their parents’ perception of appearance. Making physical appearance a topic of discussion with patients and their parents could help to create openness, because at the end it is all about perspectives.

## Data Availability

The data that support the findings of this study are available from the corresponding authors, D.G. van de Fliert and D.H.R. Kempen, upon reasonable request.
